# A Dual-Task Gait Fusion Framework for Classifying Parkinson’s Disease Severity from Wearable Sensor Data

**DOI:** 10.3390/diagnostics16152386

**Published:** 2026-07-29

**Authors:** Yinghong Yu, Shi Ye, Zihao Xia, Hao Luo, Liyong Ma

**Affiliations:** 1 Department of Control Science and Engineering, Harbin Institute of Technology, Harbin 150001, China; 23b936077@stu.hit.edu.cn; 2 School of Mechanical Engineering, Hebei University of Architecture, Zhangjiakou 075000, China; ys2025225122@hebiace.edu.cn (S.Y.); 2024225118@hebiace.edu.cn (Z.X.); maliyong@buaa.edu.cn (L.M.)

**Keywords:** Parkinson’s disease, wearable sensors, gait analysis, dual-task fusion, severity classification

## Abstract

**Objectives:** Accurate severity classification is important for sensor-based assessment of Parkinson’s disease, but overlap between adjacent stages and class imbalance can reduce model robustness. This study aimed to develop and evaluate a dual-task gait fusion framework that integrates signals collected during self-selected walking and walking with turning. **Methods:** The primary cohort comprised 87 participants with Parkinson’s disease across mild, mild-to-moderate, and moderate stages. Task-specific temporal representations were learned from the two gait conditions, concatenated and optimized using delayed class re-weighting. Performance was evaluated using subject-level stratified five-fold cross-validation with five random seeds. Generalizability was further assessed using an independent PhysioNet cohort of 93 participants with Parkinson’s disease. **Results:** The proposed method achieved an accuracy of 90.23 ± 1.27, balanced accuracy of 89.61 ± 1.13, macro F1-score of 89.10 ± 1.35, and probability-based macro AUC of 94.43 ± 1.42 on WearGait-PD. Confusion matrix and ablation analyses indicated balanced class-level performance and complementary contributions from dual-task fusion and delayed re-weighting. On the external PhysioNet cohort, accuracy, balanced accuracy, macro F1-score, and macro AUC were 84.34 ± 1.82, 82.13 ± 1.92, 81.68 ± 1.98, and 88.93 ± 2.14, respectively. **Conclusions:** Integrating turning-related gait information with self-selected walking signals improved wearable sensor-based severity classification and showed cross-dataset robustness, although validation in larger cohorts with complete severity stage coverage remains necessary.

## 1. Introduction

### 1.1. Research Background and Significance

Parkinson’s disease is a common neurodegenerative disorder characterized by bradykinesia, resting tremor, rigidity, postural instability, and gait impairment. With global population aging, its increasing prevalence has caused substantial motor dysfunction, reduced quality of life, and long-term care burden [1]. Mechanistically, Parkinson’s disease is associated with α-synuclein aggregation, mitochondrial dysfunction, neuroinflammation, and degeneration of multiple neural networks, resulting in heterogeneous and stage-dependent clinical manifestations [2]. Among these symptoms, gait impairment is particularly important because shortened stride length, reduced walking speed, increased gait variability, impaired bilateral coordination, and freezing of gait directly affect mobility and increase fall risk [3].

Clinical assessment mainly relies on physician observation and rating scales, such as the Unified Parkinson’s Disease Rating Scale and Hoehn-Yahr stage. Although these methods are widely used, they are affected by physician experience, patient status, testing environment, and assessment time, and they cannot fully capture subtle gait changes during continuous walking [4]. Wearable sensors provide a promising alternative by continuously collecting motion signals and extracting quantitative indicators such as cadence, stride length, stance phase, swing phase, gait symmetry, gait variability, and dynamic stability [5]. With the development of digital health, wearable sensor-based gait analysis has become an important pathway for constructing digital biomarkers for Parkinson’s disease [6].

Wearable gait signals can support not only patient-control discrimination but also disease staging and progression monitoring. Long-term movement tracking studies have shown that sensor-detectable motor changes may appear years before clinical diagnosis [7]. However, severity classification remains more difficult than binary diagnosis because adjacent stages often overlap, public datasets are usually small and imbalanced, and gait patterns are strongly subject-specific. Therefore, stable severity classification requires multi-source feature representation, class imbalance handling, and strict subject-level validation.

### 1.2. Wearable Sensor-Based Gait Analysis

Wearable devices can collect inertial measurement unit signals, plantar pressure, angular velocity, and acceleration. Inertial signals reflect limb swing, posture, and motion rhythm, whereas plantar pressure signals describe foot loading, stance-phase changes, and gait stability. These modalities are complementary and can provide a more comprehensive description of Parkinsonian gait abnormalities. Recent advances in instrumented wearable devices have further expanded the scope of quantitative motor assessment. Pratap et al. developed an instrumented data glove combining finger flexion and fingertip force sensing to characterize reach-to-grasp kinematics, force patterns, and inter-finger synergies. By extracting coordinated movement patterns and supporting grasp pattern classification from multisensory recordings, this work illustrates how wearable measurements can be transformed into objective, sensor-derived descriptors of human motor function [8].

Sigcha et al. [9] reviewed deep learning and wearable sensors for Parkinson’s disease diagnosis and monitoring, showing that convolutional neural networks, recurrent neural networks, and attention mechanisms can learn useful representations from motion signals. However, most existing studies still focus on binary classification, while severity grading and small-sample imbalance remain insufficiently explored. Li et al. [10] evaluated wearable sensors for early Parkinson’s disease identification and reported that inertial measurement units, smartwatches, and plantar pressure devices provide more detailed motor information than conventional clinical observation, but differences in sensor placement, acquisition tasks, feature construction, and validation strategies limit comparability and generalization.

Public datasets are essential for reproducible research. Anderson et al. [11] released WearGait-PD, which contains multi-source wearable signals from patients with Parkinson’s disease and age-matched controls under different gait tasks. The dataset supports gait abnormality and severity classification studies, but its limited sample size, class imbalance, and inter-subject variability require robust modeling strategies. The U.S. Food and Drug Administration [12] listed WearGait-PD as a regulatory science tool for evaluating wearable gait algorithms and digital biomarkers, indicating its value for research and algorithm validation. Nevertheless, strict subject-level partitioning is necessary to avoid inflated performance caused by within-subject similarity.

Voisard et al. [13] developed a clinical gait sensor dataset involving healthy individuals, neurological patients, and orthopedic patients, providing resources for cross-disease gait analysis, but its objective differs from fine-grained Parkinson’s disease severity classification. Morgan et al. [14] released a multimodal real-world mobility dataset for Parkinson’s disease, showing the value of daily-life motor data, but noise, ambiguous task boundaries, and costly annotations make severity classification more difficult.

### 1.3. Deep Learning and Multimodal Fusion

Deep learning has shifted Parkinson’s disease gait analysis from handcrafted features to end-to-end feature learning. Convolutional networks extract local temporal patterns, recurrent networks model temporal dependencies, and Transformer architectures capture long-range dynamics. These models improve representation ability but may also increase parameter size, training instability, and overfitting risk in small-sample settings. Recent research in wearable movement analysis has further demonstrated the value of multisensory fusion. Pratap et al. [15] developed Glove-Net, a hybrid CNN-BiLSTM framework that combines finger bending and fingertip force signals for grasp classification, providing complementary evidence that heterogeneous wearable signals can improve movement representation and classification.

Ji et al. [16] proposed a Wide-CNN-Transformer network for Parkinson’s disease diagnosis and severity assessment using wearable sensors, combining convolutional local feature extraction with Transformer-based long-range modeling. However, the model is complex and sensitive to parameter tuning. Ji et al. [17] further used pressure and inertial sensors for gait analysis and severity rating, showing that pressure signals reflect abnormal load transfer while inertial signals supplement limb swing and dynamic posture information. However, differences in sampling frequency, signal scale, and noise distribution make stable fusion challenging.

Jiao et al. [18] proposed a dynamic plantar pressure-based classification method combining clustering and feature selection, which improves interpretability but relies heavily on handcrafted features. Liu et al. [19] investigated population-invariant IMU-based gait biomarkers to reduce the influence of population and acquisition differences, but the work mainly focused on diagnosis rather than multi-level severity classification. Rao et al. [20] reviewed artificial intelligence methods for gait-based diagnosis and assessment of neurodegenerative diseases, but differences across diseases, tasks, and data modalities limit direct transfer to Parkinson’s disease severity classification.

Freezing of gait studies also provide useful methodological references. Sun et al. [21] combined handcrafted and deep features to predict freezing of gait, but the task focused on freezing events rather than overall severity. Khosla et al. [22] modeled transitions among normal walking, pre-freezing, and freezing states using shank acceleration data, but the method depends on specific sensor placement and annotations. Sigcha et al. [23] conducted a cross-dataset study and showed that strong within-dataset performance does not guarantee external generalization, highlighting the importance of external validation.

Borzì et al. [24] proposed real-time freezing detection using multi-head convolutional neural networks and a single inertial sensor, but one sensor may not fully capture plantar pressure and bilateral coordination. Sigcha et al. [25] applied Transformer networks to waist-worn accelerometer signals, improving long-range modeling but remaining vulnerable to small samples and class imbalance. Xu et al. [26] proposed a double-hurdle quantification model for freezing severity, extending binary detection to symptom-level assessment but still focusing on freezing rather than overall disease severity. Chan et al. [27] studied freezing detection using a single waist-worn device and showed that low device complexity benefits deployment, although sensitivity, specificity, and false alarms must be balanced.

Ghayvat et al. [28] proposed a multimodal fusion framework for pre-freezing and post-freezing intervention, showing the potential of closed-loop intervention but focusing more on control than severity classification. Xu et al. [29] proposed a graph-based multimodal fusion framework for freezing assessment, enhancing cross-modal interaction, although graph construction may increase overfitting risk in small datasets. Yang et al. [30] studied different freezing manifestations and confirmed the heterogeneity of Parkinsonian gait abnormalities, but the focus remained on freezing rather than disease stage stratification. Yang et al. [31] further showed that physiological data may improve multimodal freezing detection but may also introduce noise and redundancy.

Other modalities have also been explored. Kondo et al. [32] used video data to detect freezing of gait in clinical practice, showing that visual information can supplement wearable sensors but is affected by camera angle, occlusion, environment, and privacy. Li and Guo [33] investigated EEG-based freezing detection and prediction, extending gait analysis from a neural-signal perspective, but EEG acquisition is less convenient for daily monitoring.

### 1.4. Limitations and Research Contents

Overall, existing studies have advanced wearable gait analysis for Parkinson’s disease, but several limitations remain. First, many studies focus on binary diagnosis, while fine-grained severity classification is less explored. Second, public datasets are often small and imbalanced, causing unstable recognition of minority stages. Third, complex Transformer, graph, or multimodal models may not consistently outperform simpler architectures in small-sample medical datasets. Fourth, without subject-level partitioning, data leakage may overestimate model performance.

Pergolini et al. [34] showed that sensorized insoles can reflect center-of-pressure changes, support stability, and gait rhythm abnormalities, but insole data alone may not fully describe lower-limb swing, trunk posture, and multi-task gait changes. Gholamiangonabadi et al. [35] emphasized leave-one-subject-out cross-validation and showed that sample-level random splitting may cause subject leakage, supporting subject-level validation. Borzì et al. [36] summarized key challenges including small samples, class imbalance, data heterogeneity, and lack of external validation, indicating that severity classification should emphasize stability rather than simply increasing model complexity. Qi et al. [37] further showed that Parkinson’s disease digital biomarker research is moving toward multimodal fusion, continuous monitoring, and clinical interpretability, while reproducibility and class-level reporting remain insufficient.

Based on these limitations, this study uses the WearGait-PD dataset to develop a dual-task fusion model for automatic Parkinson’s disease severity classification. Subject-level partitioning is adopted to reduce data leakage. A dual-task feature learning framework extracts complementary representations from different gait tasks, and feature concatenation is used for fusion. To address class imbalance, delayed re-weighting enables the model to first learn general gait representations and then increase the contribution of minority classes during later training.

This study develops a clinically guided dual-task framework for subject-level Parkinson’s disease severity classification by coordinating task-specific temporal representation learning with staged imbalance-aware optimization. Self-selected walking and walking with turning are treated as paired views of the same subject, enabling the model to capture complementary information related to steady-state gait, dynamic balance, and turning-related motor adjustment. The joint representation preserves condition-specific gait information, while the staged learning strategy coordinates general representation formation with subsequent class-level refinement. Its methodological value is systematically evaluated through subject-level validation, comparisons of alternative fusion and imbalance-handling strategies, contemporary deep learning baselines, external validation, and post hoc explainability analysis.

## 2. Methods

### 2.1. Research Problem and Overall Framework

This study focuses on Parkinson’s disease severity classification based on gait signals collected by wearable sensors. A dual-task gait representation fusion model is constructed to address this problem. The proposed method takes multichannel motion signals collected under different walking conditions as input. Two task-specific temporal encoding branches are used to extract dynamic gait features from walking at a self-selected pace and walking at a self-selected pace with turning, respectively. The extracted deep features are then fused in the feature space to predict the disease severity class.

Assume that the dataset contains *N* subjects. The severity label of subject *i* is defined as:

(1) $$y_{i}\in \{1,2,3\}$$

The three severity classes were derived directly from the “Modified Hoehn & Yahr Score” provided in the clinical information file of WearGait-PD [11,38]. Specifically, H&Y scores of 1.0–1.5 were assigned to the mild class, scores of 2.0–2.5 to the mild-to-moderate (M-M) class, and scores of 3.0 or higher to the moderate (Mod.) class. Clinically, these ranges broadly represent unilateral disease with possible axial involvement, bilateral disease without established postural instability, and disease with postural instability or greater functional impairment, respectively. The labels were predefined before model development and were not derived from the wearable sensor signals or model outputs. Mild, M-M, and Mod. are operational class names used in this study to represent progressively higher H&Y ranges. For each subject, two types of gait task signals are used. The first type corresponds to walking at a self-selected pace, and the second type corresponds to walking at a self-selected pace with turning. These two signals are denoted as:

(2) $$A_{i},B_{i}\in {\mathbb{R}}^{C\times T}$$

where $A_{i}$ denotes the gait signal collected during walking at a self-selected pace, $B_{i}$ denotes the gait signal containing the turning process, *C* represents the number of sensor channels, and *T* represents the unified length of the time series.

After preprocessing, the classification objective can be formulated as:

(3) $${\hat{y}}_{i}=f({\tilde{A}}_{i},{\tilde{B}}_{i};\theta )$$

where ${\tilde{A}}_{i}$ and ${\tilde{B}}_{i}$ denote the standardized signals of the two gait tasks, f(⋅) represents the dual-task gait classification model to be optimized, *θ* denotes the model parameters, and ${\hat{y}}_{i}$ is the predicted disease severity class.

The proposed method consists of three main components. First, the two types of gait task signals are matched, normalized to a unified length, and standardized at the subject level to construct strict dual-task input samples. Second, two temporal encoding branches are employed to extract dynamic gait representations under different walking conditions. Finally, the task-related features are fused in the deep feature space, and a delayed class re-weighting strategy is introduced to optimize the classification model and reduce the influence of class imbalance during training.

**Figure 1:**
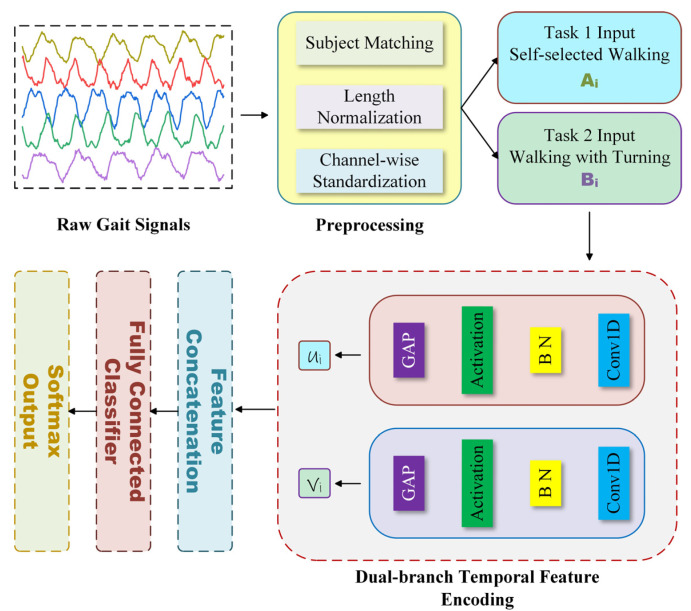
Overall architecture of the proposed dual-task gait fusion framework. Raw gait signals are first processed by subject matching, length normalization, and channel-wise standardization. The standardized self-selected walking signal and walking with turning signal are then fed into two task-specific temporal encoding branches. Each branch consists of one-dimensional convolution, batch normalization, nonlinear activation, and global average pooling. The extracted task-specific features are concatenated and passed through a fully connected classifier and Softmax layer to predict Parkinson’s disease severity.

Compared with single-task gait modeling, dual-task modeling can exploit motion information from different walking conditions. Walking at a self-selected pace mainly reflects basic gait rhythm, stability, and periodic characteristics, whereas walking with turning is more sensitive to changes in dynamic balance, postural control, and motor adjustment ability. Therefore, the two types of gait signals provide complementary information for disease severity recognition. The overall workflow of the proposed dual-task gait fusion framework is illustrated in Figure 1.

### 2.2. Subject-Level Sample Construction and Signal Standardization

To ensure reliable model evaluation, this study constructs samples at the subject level. Each sample corresponds to one subject, rather than randomly splitting multiple gait segments from the same subject into different subsets. This design prevents individual motion patterns from appearing simultaneously in the training and validation sets, thereby reducing the risk of data leakage and allowing the model performance to better reflect generalization across subjects in real clinical applications.

For each subject, the recordings from self-selected walking and walking with turning were matched according to the subject identifier. For each task, 66 signal channels were retained, including 30 IMU channels, 32 plantar-pressure channels, and four center-of-pressure channels. The IMU channels consisted of three-axis acceleration and three-axis angular velocity from the lower back, bilateral lateral shanks, and bilateral dorsal feet. The plantar-pressure channels comprised 16 pressure measurements from each foot, while the center-of-pressure channels comprised the two-dimensional coordinates for the left and right feet. Time and binary foot-contact columns were excluded. Partial missing values within each channel were filled by linear interpolation, followed by forward and backward filling at the signal boundaries; channels containing no valid values were filled with zeros. Each channel was then independently resampled to 4096 time points using one-dimensional linear interpolation over a normalized temporal axis from 0 to 1. Recordings already containing 4096 time points were retained unchanged. Thus, shorter and longer recordings were expanded or compressed using the same interpolation procedure rather than zero-padding or direct truncation.

In this study, the target sequence length was set to 4096 time points for both gait tasks. This length was selected as a fixed temporal input dimension to ensure that all subject-level dual-task samples had the same shape before model training. It also provided sufficient temporal context for the one-dimensional temporal encoders while keeping the computational cost manageable during repeated subject-level cross-validation. Thus, each subject was represented by two standardized multichannel time-series inputs with dimensions of 66 channels by 4096 time points.

To reduce the influence of differences in scale and amplitude range among different sensor channels, standardization is applied to each channel of the input signals. The standardization parameters are calculated only from the training set and then applied to the corresponding validation set, preventing statistical information from the validation set from participating in model training. For the signal value of channel *c* at time point *t*, the standardization process is defined as:

(4) $${\tilde{x}}_{c,t}=\frac{x_{c,t}-\mu_{c}}{\sigma_{c}+\varepsilon }$$

where $x_{c,t}$ denotes the original signal value, $\sigma_{c}$ and $\mu_{c}$ denote the mean and standard deviation of the *c*-th channel calculated from the training set, respectively, *ε* is a small constant used to avoid division by zero, and ${\tilde{x}}_{c,t}$ denotes the standardized signal value.

After matching, length normalization, and standardization, the dual-task sample of the *i*-th subject can be expressed as:

(5) $$D_{i}=({\tilde{A}}_{i},{\tilde{B}}_{i},y_{i})$$

where ${\tilde{A}}_{i}$ and ${\tilde{B}}_{i}$ denote the standardized self-selected walking signal and walking with turning signal, respectively, and $y_{i}$ denotes the corresponding disease severity label.

During data partitioning, a subject-level stratified cross-validation strategy is adopted. This strategy uses subjects as the basic units for data splitting and maintains, as far as possible, a similar class distribution in each fold. Compared with segment-level random splitting, subject-level splitting is more stringent. It can effectively evaluate the model’s generalization ability to unseen individuals and is more consistent with the practical use of wearable gait analysis in clinical screening and auxiliary assessment.

### 2.3. Dual-Branch Temporal Feature Encoding Network

Gait abnormalities in patients with Parkinson’s disease are not limited to local changes in signal amplitude. They are also reflected in altered gait rhythm, reduced stability, increased gait variability, and impaired postural control during turning. Therefore, this study employs a dual-branch temporal feature encoding network to model dynamic motion features from the two walking tasks separately.

For the self-selected walking signal, the first temporal encoding branch is used to extract deep gait features:

(6) $$u_{i}=E_{a}({\tilde{A}}_{i})$$

For the gait signal containing the turning process, the second temporal encoding branch is used to extract deep gait features:

(7) $$v_{i}=E_{b}({\tilde{B}}_{i})$$

where $E_{a}(\cdot )$ and $E_{b}(\cdot )$ represent the two task-specific temporal encoders, and $u_{i}$ and $v_{i}$ denote the high-level feature vectors extracted from the two gait tasks, respectively.

The two encoding branches adopt the same basic temporal modeling unit, but they process signals collected under different task conditions. This design maintains structural consistency while allowing each branch to independently learn task-specific motion representations. The self-selected walking branch focuses on basic gait rhythm and periodic characteristics, whereas the walking with turning branch pays more attention to gait adjustment, dynamic balance, and motor control changes.

Within each temporal encoding branch, one-dimensional convolution is used to capture local temporal dependencies in multichannel gait signals. The feature transformation at the *l*-th layer is defined as:

(8) $$H^{\left(l\right)}=\phi \left(BN\left(Conv1D(H^{\left(l-1\right)})\right)\right)$$

where $H^{\left(l-1\right)}$ denotes the temporal feature from the previous layer, Conv1D(⋅) denotes one-dimensional convolution, BN(⋅) denotes batch normalization, ϕ(⋅) denotes the nonlinear activation function, and $H^{\left(l\right)}$ denotes the output feature of the current layer.

Each task-specific encoder comprised four one-dimensional convolutional blocks with output channels of 128, 256, 256, and 128. The corresponding kernel sizes were 7, 5, 3, and 3, and the strides were 2, 1, 2, and 2, respectively. Symmetric padding of 3, 2, 1, and 1 samples was used for the four convolutional layers. Each convolution was followed by batch normalization and a ReLU activation function. Global average pooling was applied after the final convolutional block to generate a 128-dimensional task-specific representation. The two gait branches used the same architectural configuration but did not share trainable parameters.

By stacking multiple temporal convolutional layers, the model can gradually enlarge the receptive field and extract hierarchical features from raw sensor signals, ranging from local fluctuations to global gait patterns. Lower convolutional layers mainly capture short-term signal variations, whereas higher layers integrate motion patterns over longer temporal ranges, thereby enhancing the representation of gait characteristics related to disease severity.

To convert the temporal features generated by convolution into a fixed-dimensional subject-level representation, global average pooling is applied at the end of the encoder. Let the final temporal feature map be $H_{i}\in {\mathbb{R}}^{D\times T^{\prime }}$, where *D* denotes the number of high-level feature channels and *T′* denotes the temporal length after encoding. The global average pooling operation is given by:

(9) $$q_{i,d}=\frac{1}{T^{\prime }}\sum_{t=1}^{T^{\prime }}H_{i,d,t}$$

where $q_{i,d}$ denotes the pooled value of the *d*-th feature dimension. This operation aggregates local responses along the temporal dimension into an overall gait representation, reducing the influence of local noise and isolated abnormal time points on the final classification result.

### 2.4. Dual-Task Feature Fusion and Severity Prediction

After obtaining high-level features from the two gait tasks, feature concatenation is used to construct a comprehensive subject-level gait representation. The fusion process is defined as:

(10) $$r_{i}=[u_{i};v_{i}]$$

where $u_{i}$ and $v_{i}$ denote the feature representations generated by the two task branches, and $r_{i}$ represents the fused dual-task gait feature.

Feature concatenation is adopted instead of more complex attention-based or gated interaction modules based on the characteristics of the current task. First, Parkinson’s disease severity classification usually involves limited sample sizes and imbalanced class distributions. Although complex fusion modules may improve model capacity, they also increase the number of parameters and the difficulty of optimization. Second, the two walking tasks come from the same subject and share the same disease severity label. Feature concatenation can preserve the discriminative information from both tasks relatively completely, while allowing the classifier to learn their complementary relationship in the high-level feature space. Finally, a simple fusion structure helps improve training stability and reduce the risk of overfitting under small-sample conditions. To empirically examine the choice of fusion strategy, two additional variants were evaluated. Average fusion calculated the element-wise mean of the two 128-dimensional task-specific feature vectors. Gated fusion learned a sigmoid-activated gating vector from the concatenated task features and used it to adaptively combine the two representations. All fusion variants used the same temporal encoders, optimizer, cross-validation partitions, and random seeds, ensuring that their performance differences were attributable primarily to the fusion operation.

From a representation perspective, concatenation retains the two 128-dimensional task-specific vectors in a 256-dimensional joint space without requiring corresponding dimensions from the two gait tasks to have identical meanings. The subsequent classifier can therefore assign independent coefficients to self-selected walking and turning-related features. Element-wise averaging compresses both representations into the same dimensions and may attenuate condition-specific responses, whereas gated fusion introduces additional parameters whose estimation may be unstable in a limited cohort. This provides the methodological rationale for using concatenation as the primary fusion operation.

The fused feature vector is fed into a fully connected classification layer to obtain the discriminative scores corresponding to different severity classes:

(11) $$g_{i}=Wr_{i}+b$$

where *W* and *b* denote the weight matrix and bias term of the classification layer, respectively, and $g_{i}$ represents the class score vector of the *i*-th subject.

The class scores are then converted into class probabilities using Softmax normalization:

(12) $$P(y_{i}=k|{\tilde{A}}_{i},{\tilde{B}}_{i})=\frac{\exp(g_{i,k})}{\sum_{j=1}^{3}\exp(g_{i,j})}$$

where $P(y_{i}=k|{\tilde{A}}_{i},{\tilde{B}}_{i})$ denotes the predicted probability that the *i*-th subject belongs to the *k*-th severity class, and $g_{i,k}$ denotes the discriminative score corresponding to class *k*.

The final predicted class is determined by the maximum class probability:

(13) $${\hat{y}}_{i}=\arg\max_{k\in \{1,2,3\}}P(y_{i}=k|{\tilde{A}}_{i},{\tilde{B}}_{i})$$

Through this design, the model can jointly exploit motion information from the two gait tasks within a unified framework. Self-selected walking provides information related to basic gait rhythm, amplitude variation, and stability, whereas walking with turning further provides information associated with motor adjustment and postural control. Their fusion in the high-level feature space helps improve the model’s ability to distinguish different stages of disease severity.

### 2.5. Delayed Class Re-Weighting Strategy for Class-Imbalanced Learning

The number of subjects in different Parkinson’s disease severity stages is often imbalanced. If a standard classification loss is directly used for training, the model may be dominated by majority class samples, resulting in reduced sensitivity to minority severity classes. To alleviate this problem, this study adopts a delayed class re-weighting learning strategy. In the early training stage, standard cross-entropy loss is used so that the model can first learn stable basic gait features. After the feature space gradually becomes stable, class weights are introduced to increase the contribution of minority class samples during parameter updates.

The standard cross-entropy loss is defined as:

(14) $$L_{CE}=-\frac{1}{M}\sum_{i=1}^{M}\log P(y_{i}|{\tilde{A}}_{i},{\tilde{B}}_{i})$$

where *M* denotes the number of samples in a training batch and $P(y_{i}|{\tilde{A}}_{i},{\tilde{B}}_{i})$ denotes the predicted probability of the true class.

**Figure 2:**
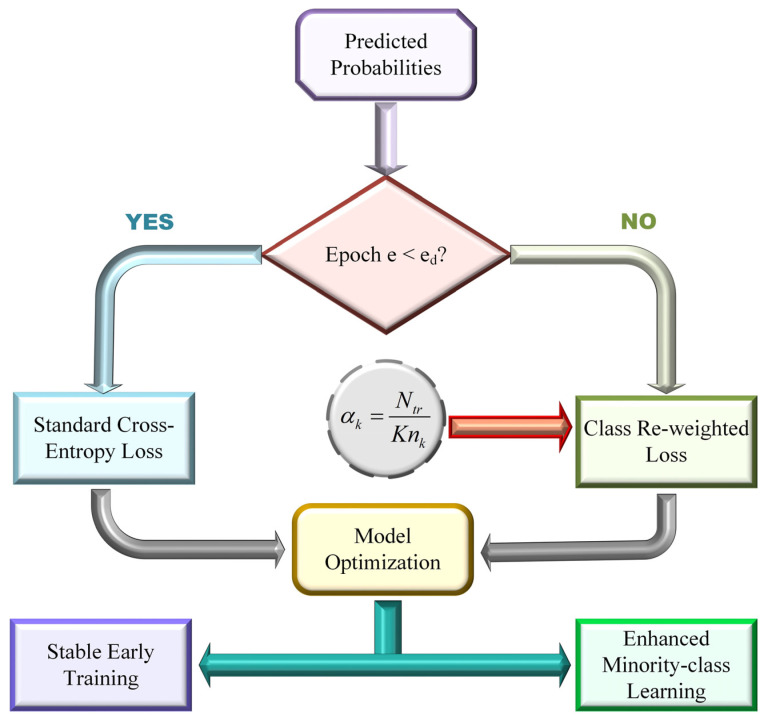
Delayed class re-weighting learning strategy. During the early training stage, the model is optimized using the standard cross-entropy loss to learn stable gait representations. After the delayed epoch threshold is reached, class weights calculated from the training-set class distribution are introduced into the loss function to enhance minority class learning. This staged optimization strategy aims to balance early representation stability and later class-level fairness.

During the class re-weighting stage, the class weight is determined according to the number of samples in each class in the training set. Let $n_{k}$ denote the number of samples in the *k*-th class, $N_{tr}$ denote the total number of training samples, and *K* denote the total number of classes. The delayed class re-weighting loss is defined as:

(15) $$\alpha_{k}=\frac{N_{tr}}{Kn_{k}},\;\;\;\;L(e)=\left\{\begin{array}{ll} L_{CE}, & e<e_{d}\\ -\frac{1}{M}\sum_{i=1}^{M}\alpha_{y_{i}}\log P(y_{i}|{\tilde{A}}_{i},{\tilde{B}}_{i}), & e\geq e_{d} \end{array}\right.$$

where $\alpha_{k}$ denotes the class weight of the *k*-th class, *e* denotes the current training epoch, and $e_{d}$ denotes the delayed epoch at which class re-weighting is activated. This weighting scheme assigns a larger loss contribution to classes with fewer samples, thereby reducing the training bias caused by class imbalance. The optimization process of the delayed class re-weighting strategy is shown in Figure 2.

In the actual training process, class weights are not enabled from the beginning. Instead, a staged optimization strategy is used. In the early stage, the model is optimized using standard cross-entropy loss to learn the overall gait distribution and basic discriminative features. In the later stage, the model is optimized using the class-weighted loss so that minority class samples have a greater influence on parameter updates. This strategy avoids optimization instability caused by excessive class weights in the early training phase and corrects the model’s bias toward majority classes in the later phase.

From an optimization perspective, class weighting changes the relative gradient contributions of different severity groups. During early training, the temporal encoders are still learning shared gait structure, and strong class-dependent scaling may make representation learning overly sensitive to the limited minority class samples. The delayed schedule therefore retains the standard objective during the initial representation learning stage and introduces class balancing progressively after the feature space becomes more stable. This representation-first and class-refinement-later strategy provides the optimization rationale for combining dual-task representation learning with staged imbalance correction.

Compared with simple oversampling methods, delayed class re-weighting does not alter the original sample distribution and therefore does not substantially increase the risk of overfitting caused by repeated use of minority class samples. Compared with applying class weighting throughout the entire training process, this strategy is more stable during early optimization. It first ensures reliable feature learning and then gradually enhances the recognition ability for minority disease stages. For Parkinson’s disease severity classification with small sample size, class imbalance, and overlapping class boundaries, this training mechanism provides a more appropriate balance between model stability and class-level fairness. For comparison with other imbalance-handling strategies, we additionally evaluated focal loss and SMOTE/augmentation-based minority class balancing. In the focal-loss variant, the standard cross-entropy objective was replaced by focal loss to increase the contribution of difficult and minority class samples during training. In the SMOTE/augmentation-based variant, synthetic minority class samples were generated only from the training set within each cross-validation fold, and the validation subjects were never used during synthetic sample generation. These variants used the same network architecture, input length, optimizer, cross-validation protocol, and random seeds as the proposed method, allowing the effect of the imbalance-handling strategy itself to be assessed.

In summary, the proposed method takes subject-level dual-task gait signals as input. Two task-specific temporal encoding branches are used to extract dynamic motion representations from self-selected walking and walking with turning, respectively, and feature concatenation is employed to obtain a comprehensive gait representation. During model optimization, a delayed class re-weighting strategy is further introduced to reduce the influence of class imbalance on severity prediction. The proposed method maintains a relatively simple structure, avoids the overfitting risk caused by overly complex models under small-sample conditions, and makes full use of dual-task gait information to improve the stability and reliability of Parkinson’s disease severity classification.

### 2.6. Post Hoc Explainability Analysis

To improve model interpretability, we performed channel-importance analysis and one-dimensional Grad-CAM visualization for the proposed dual-task fusion model. Channel importance was estimated using an occlusion-based strategy. For each trained model, one input channel was masked at a time while the remaining channels were kept unchanged, and the decrease in the predicted probability of the correct class was used as the importance score of that channel. The importance scores were then averaged across subjects, folds, and random seeds, and normalized to obtain task-specific channel-importance profiles for self-selected walking and walking with turning.

In addition, one-dimensional Grad-CAM was applied to the final convolutional feature maps of each temporal encoding branch to identify time regions that contributed most strongly to severity prediction. The gradients of the predicted class score with respect to the final temporal feature maps were used to weight the activation maps, producing a temporal saliency curve for each gait task. The resulting saliency maps were used only for post hoc interpretation and were not involved in model training or model selection.

## 3. Experimental Results

### 3.1. Experimental Setup

This study was evaluated on the WearGait-PD [11] dataset for Parkinson’s disease severity classification. Only Parkinson’s disease subjects with matched dual-task gait recordings were used. Each subject contained two walking conditions: walking at a self-selected pace and walking at a self-selected pace with turning. After preprocessing, the final experimental cohort contained 87 subjects, including 10 mild cases with H&Y scores of 1.0–1.5, 65 mild-to-moderate (M-M) cases with H&Y scores of 2.0–2.5, and 12 moderate (Mod.) cases with H&Y scores of 3.0 or higher. For each subject, both gait task signals were represented as multichannel time-series inputs with 66 channels and 4096 time points.

To reduce subject leakage, model evaluation was performed at the subject level. The experiments used five-fold cross-validation with five random seeds (42, 2024, 2025, 2026, and 3407), and the reported results were obtained under the same data partitioning and evaluation protocol for all compared models. This design ensured that samples from the same subject were not used to inflate performance across training and validation partitions.

For external validation, we used the public PhysioNet Gait in Parkinson’s Disease dataset, version 1.0.0 [39,40]. This dataset contains multichannel vertical ground reaction force recordings from patients with Parkinson’s disease and healthy controls during usual walking. The recordings were acquired from force sensors beneath the feet at 100 Hz, and the dataset also provides demographic information and clinical severity measures, including Hoehn and Yahr staging and/or UPDRS scores. To keep the severity definition consistent with the WearGait-PD experiments, Hoehn and Yahr stages 2.0–2.5 were mapped to the moderate group, and stages ≥ 3.0 were mapped to the severe group. Under this grouping rule, the external dataset contained 83 mild-to-moderate and 10 moderate Parkinson’s disease subjects, but no mild-stage subjects. Therefore, the external validation was restricted to the available mild-to-moderate and moderate severity groups.

### 3.2. Evaluation Metrics, Baselines, and Implementation Details

Model performance was evaluated using accuracy, balanced accuracy, macro F1-score, macro AUC, average precision, and macro recall. Accuracy measures the overall proportion of correctly classified samples, while balanced accuracy averages recall across classes and is therefore more suitable for imbalanced severity distributions. Macro F1-score gives equal weight to each severity class and reflects the balance between precision and recall. Macro AUC evaluates the overall class separability under a one-vs-rest setting, and average precision provides an additional probability-ranking measure under class imbalance. Macro recall was additionally reported in the ablation analysis as a class-wise sensitivity summary corresponding to the radar plot axis, whereas balanced accuracy was retained as the primary imbalance-aware classification metric.

ROC and AUC analyses were based on continuous out-of-fold prediction scores rather than hard class labels. For each random seed, the prediction vector generated for every validation subject was collected across the five subject-level folds, ensuring that each subject contributed one prediction from a model that had not been trained on that subject. For the proposed method and the neural network baselines, the continuous scores were the class probabilities generated by the Softmax layer. Random Forest and XGBoost were evaluated using their class probability outputs, whereas SVM was evaluated using continuous one-vs-rest decision scores. A one-vs-rest ROC curve was calculated separately for each severity class, and macro AUC was obtained by equally averaging the class-specific AUC values. Each seed produced one macro AUC value, and the final result was reported as mean ± standard deviation across the five random seeds. All AUC values are presented on a 0–100 scale.

The proposed method was compared with both traditional machine learning and deep learning baselines. Random Forest, SVM, and XGBoost were used as conventional baselines, whereas 1D-CNN and BiLSTM were used as deep learning baselines. All models were evaluated under the same task definition, class labels, and data partitioning strategy. This comparison was designed to test whether the proposed dual-task fusion framework improved severity classification beyond both shallow feature-based classifiers and single-stream temporal neural networks. The classical machine learning baselines were not trained using directly flattened 66 × 4096 time-series inputs. Instead, nine temporal summary statistics were extracted from each channel under each gait task: mean, standard deviation, minimum, maximum, median, 25th percentile, 75th percentile, interquartile range and root-mean-square value. This produced 66 × 9 = 594 features for each task. The features from self-selected walking and walking with turning were concatenated to form a 1188-dimensional subject-level feature vector, which was used as the common input to the classical baselines. For SVM, feature standardization was fitted exclusively on the training fold and then applied to the validation fold, whereas the tree-based baselines used the extracted features directly.

Four additional deep learning architectures were evaluated to provide a broader comparison with recent wearable time-series models: TCN, Transformer, GAT and CNN-BiLSTM. The TCN used three residual temporal convolutional blocks with a kernel size of 3 and dilation rates of 1, 2, and 4. The Transformer used two encoder layers, four attention heads, a hidden dimension of 128, and a feed-forward dimension of 256. The GAT treated the 66 sensor channels as graph nodes and used two four-head graph-attention layers to learn inter-channel relationships. The CNN-BiLSTM consisted of two one-dimensional convolutional layers with 64 and 128 filters, followed by a bidirectional LSTM with 64 units in each direction. Each model generated a 128-dimensional representation for each gait task, and the two task representations were concatenated before classification. All additional baselines used the same subject-level folds, random seeds, optimizer settings, and evaluation protocol as the proposed method.

For the proposed deep learning model, the batch size was set to 16, and the predefined training length was 200 epochs. The model was optimized using AdamW with an initial learning rate of 0.001 and a weight decay of 0.0001. The learning rate was adjusted at the end of each epoch using cosine-annealing scheduling and gradually decreased to 0.000001 over the 200 training epochs. The schedule was predefined and did not depend on validation-set performance. Each task-specific encoder generated a 128-dimensional representation. After concatenation, the resulting 256-dimensional feature vector was processed by a 256-dimensional fully connected layer followed by ReLU activation. Dropout with a rate of 0.30 was applied after this fusion layer and immediately before the final classification layer. No dropout was used within the convolutional encoding blocks. Mixed-precision training was enabled during model optimization.

A single hyperparameter configuration was specified before the repeated cross-validation experiments and was applied unchanged across all folds and random seeds. No fold-specific hyperparameter search was performed using the outer validation subjects. The fixed configuration included a batch size of 16, a maximum training length of 200 epochs, an initial learning rate of 0.001, a weight decay of 0.0001, 128-dimensional task representations, a 256-dimensional fusion hidden layer, a dropout rate of 0.30, and a predefined delayed class re-weighting schedule that was applied consistently across all folds and random seeds.

Within each outer cross-validation fold, signal-standardization parameters, class weights, and model parameters were estimated exclusively from the corresponding training subjects. The outer validation fold was used only for final performance evaluation and did not influence hyperparameter selection, learning-rate adjustment, early stopping, or checkpoint selection. Each deep learning model was trained for the predefined 200 epochs, and the final-epoch checkpoint was used to calculate the reported performance.

To evaluate deployment relevance, we further quantified the computational complexity of the deep learning models. Trainable parameter count was calculated as the total number of learnable model parameters. FLOPs were estimated for one subject-level forward pass with batch size 1, using paired dual-task inputs with dimensions of 66 channels and 4096 time points for each gait task. If the profiling tool reported multiply-accumulate operations, one MAC was converted to two FLOPs. Model-only inference time was measured with batch size 1 after 50 warm-up iterations and 500 timed forward passes on an NVIDIA GeForce RTX 5070 GPU. Inference time is reported as mean ± standard deviation in milliseconds per subject-level sample, excluding disk I/O.

To address class imbalance, delayed re-weighting was introduced during training. The model first used the standard classification loss to learn stable gait representations. Class re-weighting was introduced after the initial representation learning stage and was progressively strengthened during the subsequent training stage. The same predefined schedule was applied across all cross-validation folds and random seeds. This design was intended to avoid unstable early optimization caused by excessive minority class weighting, while still improving class-level fairness in the later training stage.

To evaluate whether the selected temporal input length affected model performance, we further conducted a target sequence length sensitivity analysis. The proposed model was trained and evaluated using target lengths of 2048, 4096, and 8192 time points. For this analysis, the model architecture, subject-level five-fold cross-validation protocol, five random seeds, optimizer, training epochs, delayed re-weighting schedule, and evaluation metrics were kept unchanged. All sensitivity-analysis results are reported as mean ± standard deviation across five seed-level cross-validation averages, with each seed-level value averaged across the five subject-level folds.

For each of the five random seeds, subject-level stratified five-fold cross-validation was performed. Within each seed, identical fold assignments were used for all models and ablation variants to ensure paired comparisons. Each performance metric was first averaged across the five folds, producing one seed-level cross-validation result. The reported mean and standard deviation were subsequently calculated from the five seed-level results. Therefore, the reported variability reflects differences across repeated seed-level cross-validation results rather than treating the 25-fold seed evaluations as independent observations.

To further characterize the uncertainty of model performance, two-sided 95% confidence intervals were estimated from the five seed-level cross-validation averages. Student’s t distribution was used because only five repeated estimates were available. Class-specific recall was evaluated using the same five-seed procedure, and its two-sided 95% confidence interval was calculated from the five seed-level estimates using Student’s *t* distribution. The resulting intervals describe the variability observed across repeated cross-validation evaluations and complement the reported mean and standard deviation. Paired statistical comparisons were additionally performed between the proposed method and the two strongest baselines, CNN-BiLSTM and Transformer. For each performance metric, the results were paired using the identical subject-level fold and random seed. Because estimates from repeated cross-validation folds are correlated, statistical significance was assessed using a corrected repeated cross-validation *t*-test. Holm’s procedure was applied across the eight comparisons involving two baseline models and four performance metrics. An adjusted *p*-value below 0.05 was considered statistically significant.

For the external validation experiment, model development and hyperparameter selection were performed using only the WearGait-PD data. The PhysioNet dataset was used as an independent external test cohort and was not used for model selection. Because the PhysioNet recordings are based on plantar force sensors rather than wearable IMU signals, the external validation was interpreted as a cross-dataset robustness test under a different sensor modality and walking protocol. Performance on the external cohort was reported using the same evaluation metrics as the internal experiments, including accuracy, balanced accuracy, macro F1-score, macro AUC, and macro recall.

### 3.3. Overall Performance Comparison

**Figure 3:**
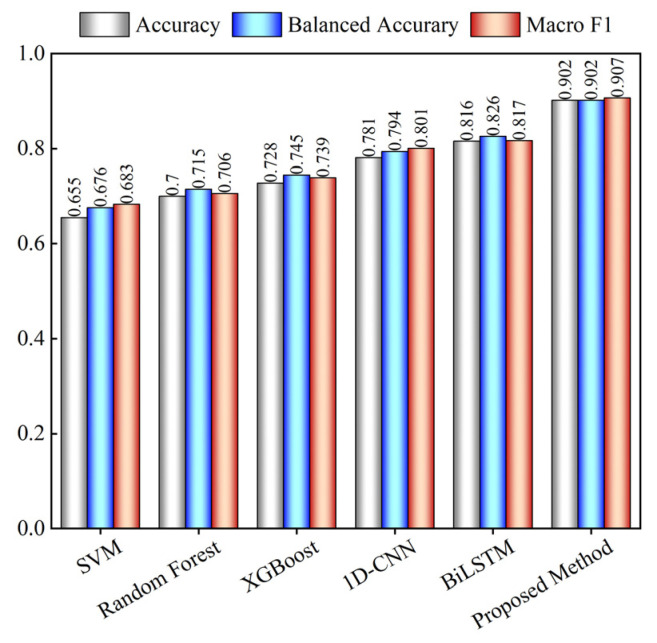
Performance comparison of the six classification models. Accuracy, balanced accuracy, and macro F1-score are reported for SVM, Random Forest, XGBoost, 1D-CNN, BiLSTM, and the proposed method. Complete comparisons with TCN, Transformer, GAT, and CNN-BiLSTM are provided in Table 1.

Table 1 reports mean ± standard deviation across five seed-level cross-validation averages, while Figure 3 provides a visual comparison of representative models. The proposed method achieved an accuracy of 90.23 ± 1.27 (95% CI: 88.65–91.81), a balanced accuracy of 89.61 ± 1.13 (95% CI: 88.21–91.01), a macro F1-score of 89.10 ± 1.35 (95% CI: 87.42–90.78), and a macro AUC of 94.43 ± 1.42 (95% CI: 92.67–96.19). The corresponding confidence intervals for CNN-BiLSTM, the strongest baseline, were 85.98–88.82, 85.14–89.44, 84.99–88.87, and 91.13–94.19, respectively. These results indicate that the proposed method maintained consistently high mean performance across repeated evaluations.

The comparative results show that the recently added deep learning architectures generally outperformed the conventional baselines and the basic 1D-CNN and BiLSTM models. CNN-BiLSTM was the strongest baseline, achieving an accuracy of 87.40 ± 1.14, a balanced accuracy of 87.29 ± 1.73, a macro F1-score of 86.93 ± 1.56, and a macro AUC of 92.66 ± 1.23. The proposed method achieved the best overall results, improving over CNN-BiLSTM by 2.83 points in accuracy, 2.32 points in balanced accuracy, 2.17 points in macro F1-score, and 1.77 points in macro AUC.

Paired statistical comparisons further demonstrated the consistency of these improvements across matched folds and random seeds. Compared with CNN-BiLSTM, the Holm-adjusted *p*-values for accuracy, balanced accuracy, macro F1-score, and macro AUC were 0.012, 0.029, 0.037, and 0.024, respectively. Compared with Transformer, the corresponding adjusted *p*-values were 0.004, 0.013, 0.008, and 0.019. All comparisons remained statistically significant after correction for repeated cross-validation and multiple testing.

### 3.4. ROC and Class-Level Performance Analysis

**Figure 4:**
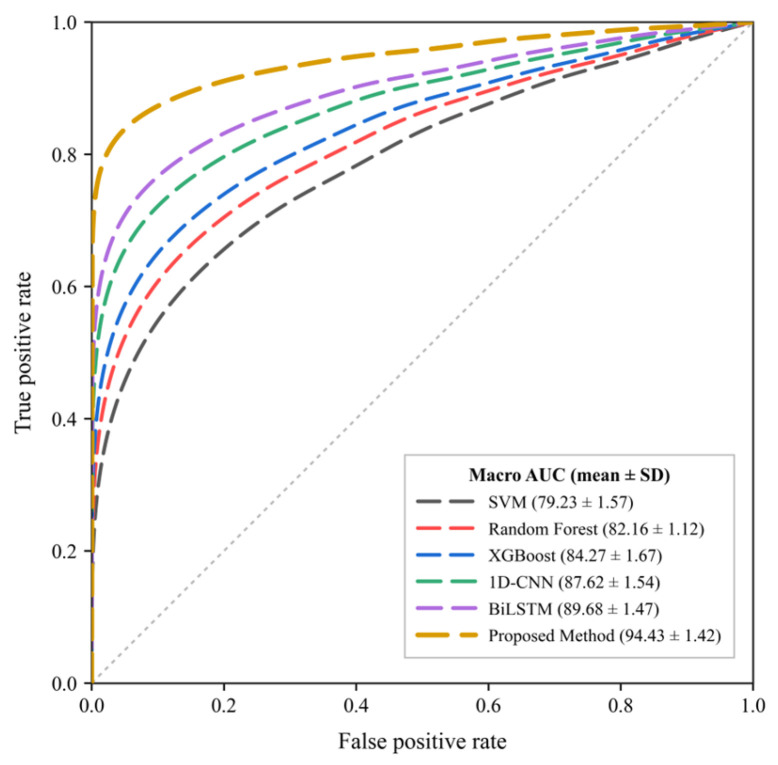
Probability-based macro-average ROC curves for six representative classification models. For each random seed, one-vs-rest ROC curves were calculated from continuous out-of-fold class probabilities or decision scores, and the class-specific curves were equally averaged to obtain the macro-average curve. The legend reports macro AUC as mean ± standard deviation across five random seeds on a 0–100 scale. The grey diagonal represents chance-level discrimination.

Figure 4 presents the probability-based macro-average ROC curves for six representative classification models. The proposed method achieved the highest macro AUC of 94.43 ± 1.42, followed by BiLSTM at 89.68 ± 1.47, 1D-CNN at 87.62 ± 1.54, XGBoost at 84.27 ± 1.67, Random Forest at 82.16 ± 1.12, and SVM at 79.23 ± 1.57. All ROC curves were constructed from continuous out-of-fold class probabilities or decision scores rather than hard predicted labels. The higher macro AUC of the proposed method indicates stronger overall probability-ranking performance across the three severity classes and complements the hard-label classification metrics reported in Table 1.

**Figure 5:**
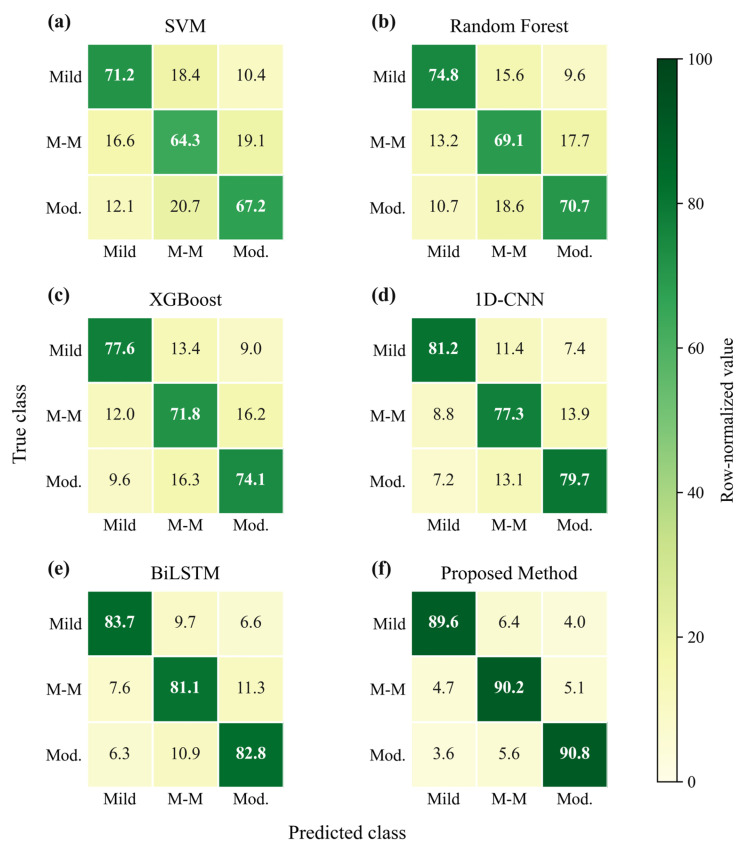
Row-normalized confusion matrices of six classification models. (**a**) SVM, (**b**) Random Forest, (**c**) XGBoost, (**d**) 1D-CNN, (**e**) BiLSTM, and (**f**) the proposed method. Rows indicate the true severity classes and columns indicate the predicted classes. All panels use a common 0–100 color scale. The operational class labels correspond to H&Y scores of 1.0–1.5 for mild, 2.0–2.5 for mild-to-moderate (M-M), and ≥3.0 for moderate (Mod.).

Class-level performance was further examined using confusion matrices (Figure 5). The proposed method (Figure 5f) produced the strongest diagonal responses across all three severity classes. Across the five seed-level cross-validation averages, class-specific recall was 89.60 ± 4.17% (95% CI: 84.42–94.78%) for Mild, 90.20 ± 1.17% (95% CI: 88.74–91.66%) for M-M, and 90.80 ± 3.32% (95% CI: 86.68–94.92%) for Mod. In contrast, BiLSTM achieved recalls of 83.7%, 81.1%, and 82.8%, respectively, and the traditional models showed larger off-diagonal errors. The largest off-diagonal error of the proposed method was 6.4%, whereas the corresponding maximum off-diagonal errors were 11.3% for BiLSTM, 13.9% for 1D-CNN, and 20.7% for SVM. These results indicate that the performance gain was not concentrated in a single class but was distributed across different severity stages. The reduced confusion between neighboring stages also supports the value of combining self-selected walking and turning-related gait information.

### 3.5. Feature Distribution Visualization

**Figure 6:**
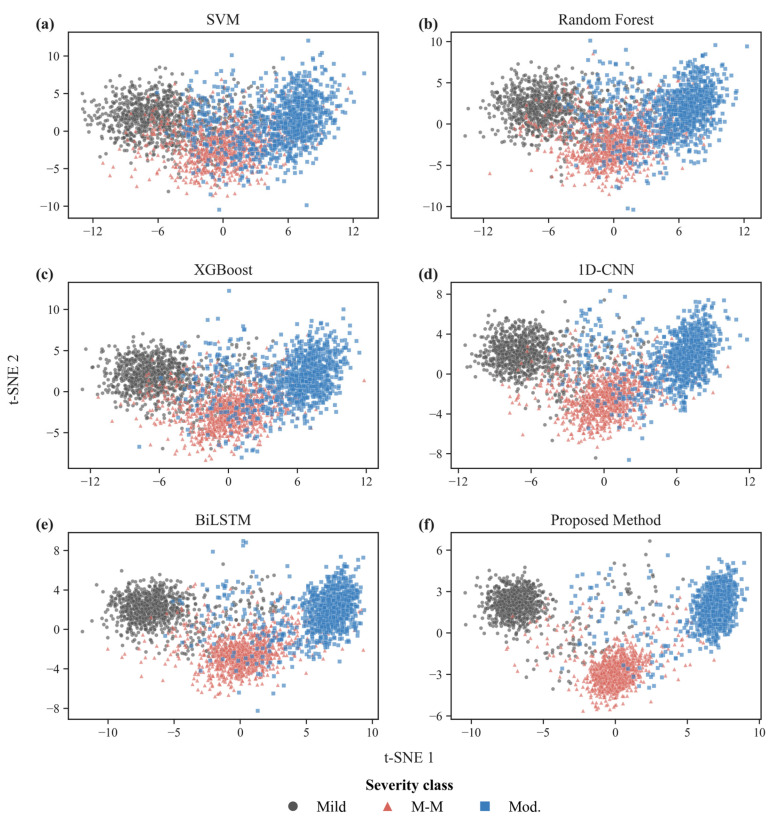
t-SNE visualization of feature distributions from six classification models. (**a**) SVM, (**b**) Random Forest, (**c**) XGBoost, (**d**) 1D-CNN, (**e**) BiLSTM, and (**f**) the proposed method. Each point represents a sample, and consistent colors and marker shapes indicate the three Parkinson’s disease severity classes across all panels. The operational class labels correspond to H&Y scores of 1.0–1.5 for mild, 2.0–2.5 for mild-to-moderate (M-M), and ≥3.0 for moderate (Mod.).

The t-SNE visualization provides a representation-level explanation for the classification results (Figure 6). Compared with the baseline models, the proposed method showed clearer separation among the three severity groups, especially for the transition between the M-M and Mod. classes. The traditional machine learning models showed more substantial overlap among classes, and the single-stream deep models still retained a mixed central region. In the proposed method, the Mild and Mod. samples formed more distinct regions, while the M-M group was located between them, which is consistent with the ordinal nature of disease severity. Because t-SNE is a qualitative visualization rather than a quantitative classifier, this figure should be interpreted as supporting evidence for feature separability rather than as standalone proof of model performance.

### 3.6. Training Convergence Analysis

**Figure 7:**
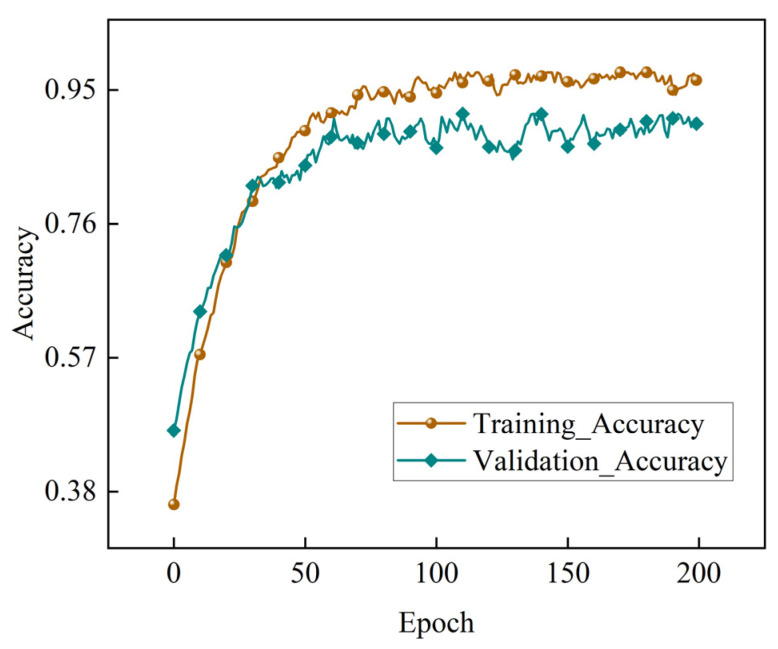
Training and validation accuracy curves of the proposed method. The curves show the optimization process over 200 epochs.

The training accuracy curves show that the proposed model reached stable optimization within 200 epochs (Figure 7). Training accuracy increased rapidly during the early epochs and then stabilized at a high level, with a final value of 0.964 and a best value of 0.975. Validation accuracy followed a similar upward trend and stabilized around 0.904 over the final 20 epochs, with a final value of 0.902 and a best value of 0.916. The gap between training and validation accuracy indicates that some generalization gap remains, which is expected in a small-sample medical dataset, but the validation curve did not collapse after convergence. This pattern suggests that the training process was stable and that the proposed model maintained useful predictive performance on validation data.

The epoch-wise validation curves were generated only for post hoc visualization of convergence behavior. The highest validation accuracy and lowest validation loss were not used for hyperparameter adjustment, early stopping, or checkpoint selection, and all comparative results were obtained from the predefined final-epoch checkpoint.

**Figure 8:**
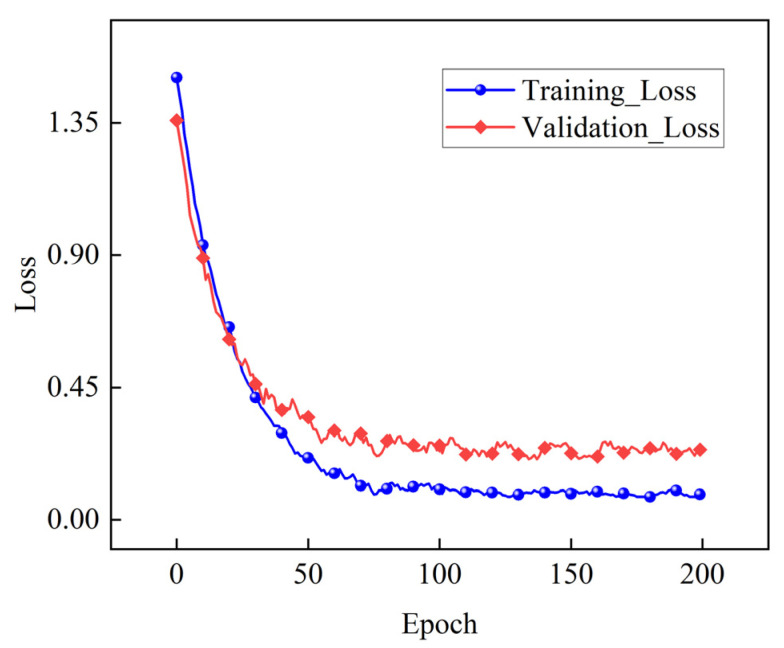
Training and validation loss curves of the proposed method. The decreasing loss values indicate stable convergence during model training.

The loss curves provide complementary evidence for the convergence behavior (Figure 8). Training loss decreased continuously and reached a final value of 0.086, while validation loss decreased to a stable range with a final value of 0.238. Over the final 20 epochs, the average training loss and validation loss were approximately 0.088 and 0.237, respectively. The validation loss remained higher than the training loss, which again reflects the difficulty of generalizing under limited sample size and inter-subject variability. However, the validation loss did not show a sustained upward trend after the model converged, suggesting that the optimization process did not suffer from severe late-stage overfitting. Together with the accuracy curves, this supports the reliability of the reported comparison results.

### 3.7. Ablation Study

To examine the contribution of each design component, several ablation variants were evaluated. “SelfPace only” used only the self-selected walking task, whereas “matTURN only” used only the walking with turning task. “Dual-task fusion” combined the two gait tasks without delayed re-weighting. “Average fusion” calculated the element-wise mean of the two task-specific feature vectors, whereas “Gated fusion” learned an adaptive sigmoid gate to regulate the relative contributions of the two gait tasks. “Class weight from start” applied class weighting from the beginning of training. “Focal loss fusion” replaced the standard cross-entropy loss with focal loss for the dual-task fusion model. “SMOTE/augmentation fusion” used training-set-only synthetic minority class augmentation to alleviate class imbalance. “Sampler fusion” used sampling-based class balancing for the dual-task fusion model. “DRW sampler fusion” combined delayed re-weighting with sampling-based balancing. These variants were compared with the full proposed method to assess whether dual-task fusion and delayed class re-weighting jointly improved severity classification.

**Figure 9:**
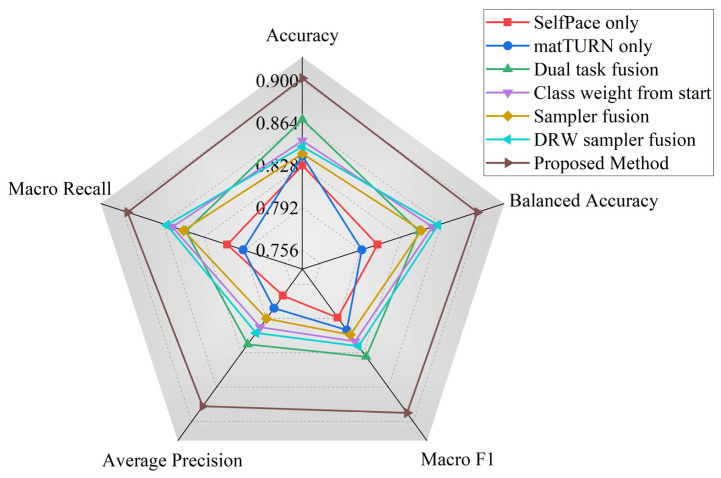
Ablation analysis of the proposed dual-task fusion framework. The figure compares single-task inputs, simple dual-task fusion, focal loss, SMOTE/augmentation-based balancing, sampling-based balancing, delayed re-weighting variants, and the complete proposed method across multiple evaluation metrics. The complete framework achieves the most balanced overall profile, supporting the contribution of both dual-task gait fusion and delayed re-weighting. Complete numerical comparisons, including average fusion, gated fusion, focal loss, and SMOTE/augmentation, are provided in Table 2.

The ablation results demonstrate the contribution of the main design components (Table 2 and Figure 9). Using either gait task alone produced lower performance than dual-task fusion, indicating that self-selected walking and walking with turning captured complementary information related to Parkinson’s disease severity. The self-selected walking branch achieved an accuracy of 0.828 and a macro F1-score of 0.791, whereas the turning-task branch achieved an accuracy of 0.836 and a macro F1-score of 0.804. Their combination improved the accuracy to 0.867 and the macro F1-score to 0.832, suggesting that the two task conditions provided non-redundant temporal cues.

The comparison among class-balancing strategies further shows that the timing of re-weighting was important. Applying class weights from the beginning increased the class-sensitivity-oriented metrics compared with simple dual-task fusion, but it reduced macro F1 and average precision compared with the complete framework, suggesting that strong early re-weighting may disturb representation learning under the small-sample WearGait-PD setting. Similarly, sampling-based balancing and DRW sampler fusion did not match the full proposed method. In contrast, the complete dual-task fusion model with delayed re-weighting achieved the best overall performance, with an accuracy of 0.902, a balanced accuracy of 0.896, a macro F1-score of 0.891, an average precision of 0.884, and a macro recall of 0.895. These results indicate that complementary dual-task gait information and delayed class re-weighting jointly contributed to more stable and balanced severity classification.

Among the evaluated fusion strategies, gated fusion achieved better overall performance than average fusion, indicating that adaptive regulation of the two task representations was more effective than direct element-wise averaging. Gated fusion achieved an accuracy of 87.81 ± 1.29% and a balanced accuracy of 86.48 ± 1.35%, whereas average fusion achieved 85.42 ± 1.38% and 83.91 ± 1.52%, respectively. Nevertheless, the complete framework combining feature concatenation with delayed re-weighting achieved the best overall results. Focal loss and SMOTE/augmentation improved several class-sensitive metrics compared with simple dual-task fusion, but neither achieved the overall balance of the proposed method. These comparisons further support the selected fusion and class-imbalance-handling strategies.

### 3.8. Sensitivity Analysis of Target Sequence Length

To examine the robustness of the proposed framework to temporal input length, we compared target lengths of 2048, 4096, and 8192 time points using the same subject-level cross-validation protocol and training settings. As shown in Table 3, the 2048-point setting achieved an accuracy of 88.72 ± 1.35, whereas increasing the input length to 4096 improved the accuracy to 90.23 ± 1.27. Further increasing the sequence length to 8192 resulted in an accuracy of 89.17 ± 1.50 without providing an additional performance gain. Similar patterns were observed for balanced accuracy, macro F1-score, macro AUC, average precision, and macro recall. Therefore, 4096 time points were retained because this setting provided the best overall balance between classification performance and computational cost.

### 3.9. External Validation on an Independent Parkinson’s Gait Dataset

To assess the generalizability of the proposed method beyond the WearGait-PD cohort, we performed an external validation experiment on the public PhysioNet Gait in Parkinson’s Disease dataset. Under the same Hoehn and Yahr-based severity mapping used in the internal cohort, this external dataset contained 83 mild-to-moderate and 10 moderate Parkinson’s disease subjects, but no mild-stage subjects. Therefore, the external experiment was used to evaluate cross-dataset robustness for the available severity categories rather than full three-class severity classification. External validation results were summarized as mean ± standard deviation across repeated model training seeds. As shown in Table 4, the proposed framework achieved an external accuracy of 84.34 ± 1.82, balanced accuracy of 82.13 ± 1.92, macro F1-score of 81.68 ± 1.98, macro AUC of 88.93 ± 2.14, and macro recall of 82.12 ± 1.71.

### 3.10. Computational Complexity and Inference Efficiency

To assess the feasibility of the proposed method for clinical and wearable gait-analysis scenarios, we quantified model size, computational cost, and inference latency. As shown in Table 5, the proposed model contained 1.12 M trainable parameters and required 2.86 GFLOPs for one subject-level dual-task forward pass. With batch size 1, the model achieved an average inference time of 2.91 ± 0.17 ms per subject-level sample on an NVIDIA GeForce RTX 5070 GPU. These results provide a quantitative reference for potential deployment in offline clinical assessment or wearable sensor analysis pipelines.

### 3.11. Channel Importance and Grad-CAM Visualization

**Figure 10:**
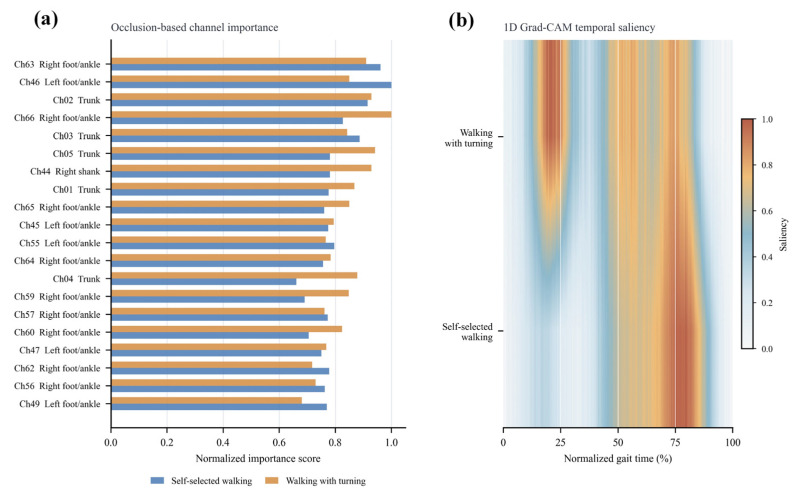
Explainability analysis of the proposed dual-task fusion model. (**a**) Channel importance profiles estimated by occlusion-based analysis for self-selected walking and walking with turning. Higher values indicate a larger decrease in correct class confidence after masking the corresponding input channel. (**b**) One-dimensional Grad-CAM visualization of temporal regions contributing to severity prediction. Warmer colors indicate stronger Grad-CAM saliency in the corresponding temporal segments. The visualization suggests that foot/ankle, shank, trunk-related, and turning-related temporal patterns contributed to the model’s severity classification.

To examine whether the proposed model relied on clinically meaningful gait information, we performed channel-importance and one-dimensional Grad-CAM analyses. As shown in Figure 10a, the highly ranked channels were mainly distributed in foot/ankle, shank, and trunk-related sensor signals, and the channel-importance profiles showed task-dependent differences between self-selected walking and walking with turning. This suggests that the two gait tasks provided complementary information for severity prediction rather than one task uniformly dominating the other. This pattern is consistent with the clinical relevance of distal lower-limb movement, trunk stability, and postural control in Parkinsonian gait.

The Grad-CAM visualization further showed that the model focused on discriminative temporal regions rather than uniformly weighting the entire gait sequence. As shown in Figure 10b, salient regions were observed in both gait tasks, with stronger and more extended responses in the walking with turning condition, especially in the middle-to-late portions of the normalized sequence. These findings support the clinical plausibility of the learned representations and suggest that the proposed framework captured gait features related to rhythm stability, dynamic balance, and turning-related motor adjustment.

## 4. Discussion

This study addressed the challenge of classifying Parkinson’s disease severity from wearable gait signals under a small, imbalanced, and subject-specific data regime. The main finding is that combining self-selected walking with walking that includes turning provided a more informative representation than using either task alone. In the reported evaluation, the proposed fusion framework achieved the best overall performance among the compared machine learning and deep learning baselines, with high accuracy, balanced accuracy, macro F1-score, and probability-based macro AUC. More importantly, the confusion matrix analysis showed balanced recalls across mild, mild-to-moderate, and moderate stages, suggesting that the performance gain was not driven only by the majority class.

The benefit of combining the two gait tasks is clinically and biomechanically plausible. Self-selected walking mainly reflects baseline gait rhythm, stride regularity, and steady-state locomotor control. In contrast, walking with turning introduces additional demands on dynamic balance, axial control, postural adjustment, and gait transition. These turning-related demands may expose motor impairments that are less obvious during straight walking. Therefore, the two task conditions provide complementary information for severity classification. The feature visualization further supports this interpretation: the proposed method produced a clearer separation among the three severity groups, while the mild-to-moderate group remained located between the two endpoint stages, which is consistent with the ordinal nature of disease progression. The added explainability analysis further supports this interpretation, as the channel-importance and Grad-CAM results indicated that the model emphasized lower-limb, trunk-related, and turning-related temporal patterns that are clinically relevant to gait rhythm, postural control, and dynamic balance impairment in Parkinson’s disease. By linking model predictions to anatomically and temporally interpretable gait patterns, these analyses improve model transparency and may facilitate clinician review of model outputs in future decision-support settings.

The delayed class re-weighting strategy also contributed to the final performance profile. In this dataset, the mild-to-moderate group was much larger than the mild and moderate groups, making the classifier vulnerable to majority class bias. Applying class weights from the beginning can increase the influence of minority classes, but it may also distort early representation learning when the feature space is still unstable. The delayed strategy used in this work first allowed the network to learn common gait patterns and then increased the contribution of minority stage samples in the later training phase. The ablation results support this design choice, because the complete framework achieved a more balanced overall profile than simple dual-task fusion, class weighting from the start, and sampling-based balancing variants.

Another implication is that a relatively simple fusion design can be effective in small-sample medical sensor datasets. More complex attention, graph, or Transformer-based fusion modules may increase modeling capacity, but they also introduce additional parameters and a higher risk of overfitting. The proposed model used task-specific temporal encoders followed by feature concatenation, which preserved information from both walking conditions while keeping the fusion mechanism stable. This design is aligned with the practical constraints of wearable gait analysis, where sample size, inter-subject variability, and class imbalance often matter as much as model capacity. The computational profiling results further support this interpretation, showing that the proposed model required 1.12 M parameters, 2.86 GFLOPs, and 2.91 ± 0.17 ms inference time per subject-level sample on an RTX 5070 GPU. Accordingly, the methodological advance should be interpreted as the coordinated use of clinically distinct gait tasks, information-preserving feature fusion, and staged imbalance correction under strict subject-level evaluation. The fusion- and imbalance-handling ablations indicate that the performance gain was associated with their combined configuration rather than simply increasing network depth or model complexity.

The study also emphasizes the importance of subject-level validation. Wearable gait recordings from the same participant can contain strong individual motion signatures. If segments from one subject are randomly distributed across training and validation sets, the model may learn subject identity rather than disease severity, leading to inflated performance. By constructing subject-level samples and applying subject-level cross-validation, the present evaluation provides a stricter estimate of generalization to unseen individuals. Together with the independent PhysioNet evaluation, this subject-level protocol provides complementary evidence of robustness within and across datasets, while broader multi-center validation remains necessary.

Beyond disease assessment and monitoring, wearable gait analytics may also provide quantitative inputs for intelligent rehabilitation systems. Narayan et al. [41] developed an “assist-as-needed” framework for a pediatric lower-limb exoskeleton by integrating a neuro-fuzzy variable admittance model with robust adaptive backstepping sliding-mode gait-tracking control. The framework adjusts robotic assistance according to subject-exoskeleton interaction, supporting compliant gait tracking, active user participation, and individualized assistance. These developments suggest that wearable sensing and gait modeling may contribute to a broader clinical pathway encompassing severity assessment, longitudinal monitoring, rehabilitation planning and adaptive robotic assistance.

Despite these strengths, several limitations should be acknowledged. First, the final cohort contained only 87 Parkinson’s disease subjects, with a highly imbalanced class distribution. This limits the statistical certainty of class-level estimates, especially for the mild and moderate groups. To reduce overinterpretation of point estimates, we reported mean ± standard deviation, *t*-based 95% confidence intervals, and corrected paired statistical comparisons with the strongest baseline models. Nevertheless, the limited number of mild and moderate subjects remains an important limitation for estimating class-specific performance. Although the hyperparameters were fixed before repeated cross-validation and the outer validation folds were isolated from parameter optimization and model selection, the limited cohort size still increases the risk of model-specific overfitting. Future studies should employ nested cross-validation or an independent development cohort for systematic hyperparameter optimization while preserving a separate external cohort for final performance evaluation. Second, although we added an external validation experiment using the PhysioNet Gait in Parkinson’s Disease dataset, the external cohort differed from WearGait-PD in sensor modality and walking protocol and did not include mild-stage Parkinson’s disease subjects under our Hoehn and Yahr-based grouping rule. Therefore, the external validation supports cross-dataset robustness for the available mild-to-moderate and moderate groups, but further validation in larger multi-center cohorts with complete severity coverage remains necessary. Third, the task setting used two specific walking conditions from WearGait-PD; the model may require additional validation before being applied to free-living gait, different sensor placements, different sampling protocols, or other clinical populations. Fourth, the current framework focuses mainly on sensor-derived gait signals and does not explicitly integrate medication state, disease duration, UPDRS scores, cognitive status, or other clinical variables that may affect gait severity.

The ROC, t-SNE, and convergence analyses should also be interpreted as supportive rather than definitive evidence. The ROC analysis based on continuous out-of-fold probability outputs provides a threshold-independent assessment of class separability. Although AUC calculation does not require calibrated probabilities, probability calibration and clinically relevant decision thresholds should be further evaluated in larger independent cohorts before clinical deployment. Similarly, t-SNE is useful for visualizing representation structure, but it does not provide quantitative proof of generalization. Future work should therefore validate the model on larger multi-center datasets, examine robustness under different walking tasks and sensor configurations, and evaluate whether adding clinical metadata or interpretable gait biomarkers can improve both accuracy and clinical trust. Longitudinal validation would be particularly valuable for determining whether the fused gait representation can track progression over time rather than only classify cross-sectional severity stages.

## 5. Conclusions

This paper proposed a dual-task gait fusion framework for classifying Parkinson’s disease severity from wearable sensor data. By extracting task-specific temporal representations from self-selected walking and walking with turning, and by introducing delayed class re-weighting during training, the method aimed to address overlapping gait patterns and class imbalance in small-sample severity classification.

The experimental results indicate that the proposed framework outperformed the evaluated traditional machine learning and deep learning baselines under subject-level cross-validation. The overall comparison, class-level confusion matrices, feature visualization, and ablation study jointly support the value of combining complementary gait tasks and delaying class re-weighting until after early representation learning. These findings suggest that turning-related gait information can strengthen wearable sensor-based assessment of Parkinson’s disease severity.

The current evidence remains bounded by the size and composition of the WearGait-PD cohort and by the limited severity coverage and different sensor modality of the external validation dataset. Therefore, the method should be regarded as a promising auxiliary modeling framework rather than a replacement for clinical staging. Future studies should test the framework in larger and more diverse cohorts, evaluate longitudinal progression monitoring, and integrate interpretable gait and clinical features to improve reliability for practical clinical use.

## Figures and Tables

**Table 1:** Performance comparison of ten classification models. Values are reported as mean ± standard deviation of five seed-level cross-validation averages, with each seed-level value averaged across five subject-level folds. All metrics are presented on a 0–100 scale. The final two rows report Holm-adjusted *p*-values obtained using corrected repeated cross-validation paired tests.

Model/Comparison	Accuracy	Balanced Accuracy	Macro F1	Macro AUC
SVM	65.49 ± 1.53	67.57 ± 1.41	68.32 ± 1.38	79.23 ± 1.57
Random Forest	69.98 ± 1.18	71.53 ± 1.56	70.64 ± 1.19	82.16 ± 1.12
XGBoost	72.78 ± 1.45	74.50 ± 1.51	73.92 ± 1.56	84.27 ± 1.67
1D-CNN	78.08 ± 1.26	79.40 ± 1.45	80.11 ± 1.35	87.62 ± 1.54
BiLSTM	81.63 ± 1.62	82.53 ± 1.28	81.72 ± 1.52	89.68 ± 1.47
TCN	84.72 ± 1.26	84.91 ± 1.64	84.30 ± 1.11	90.14 ± 1.59
Transformer	85.94 ± 1.55	86.16 ± 1.36	85.67 ± 1.46	91.79 ± 1.66
GAT	84.38 ± 1.06	84.86 ± 1.65	83.90 ± 1.14	90.84 ± 1.51
CNN-BiLSTM	87.40 ± 1.14	87.29 ± 1.73	86.93 ± 1.56	92.66 ± 1.23
Proposed Method	90.23 ± 1.27	89.61 ± 1.13	89.10 ± 1.35	94.43 ± 1.42
Holm-adjusted *p*: Proposed vs. CNN-BiLSTM	0.012	0.029	0.037	0.024
Holm-adjusted *p*: Proposed vs. Transformer	0.004	0.013	0.008	0.019

**Table 2:** Ablation results of different model variants. Values are reported as mean ± standard deviation of five seed-level cross-validation averages, with each seed-level value averaged across five subject-level folds. All metrics are presented on a 0–100 scale.

Ablation Variant	Accuracy	Balanced Accuracy	Macro F1	Average Precision	Macro Recall
SelfPace only	82.86 ± 1.58	80.78 ± 1.46	79.11 ± 1.43	76.87 ± 1.62	80.66 ± 1.23
matTURN only	83.69 ± 1.61	79.33 ± 1.24	80.45 ± 1.17	78.16 ± 1.50	79.13 ± 1.59
Dual-task fusion	86.71 ± 1.56	84.49 ± 1.61	83.20 ± 1.72	81.92 ± 1.31	84.69 ± 1.69
Class weight from start	84.90 ± 1.18	85.64 ± 1.15	81.65 ± 1.47	80.11 ± 1.54	85.59 ± 1.77
Average fusion	85.42 ± 1.38	83.91 ± 1.52	82.67 ± 1.44	81.30 ± 1.61	83.88 ± 1.55
Gated fusion	87.81 ± 1.29	86.48 ± 1.35	85.76 ± 1.41	84.63 ± 1.47	86.45 ± 1.37
Focal loss fusion	86.34 ± 1.42	86.89 ± 1.36	85.12 ± 1.48	83.84 ± 1.51	86.87 ± 1.39
SMOTE/augmentation fusion	85.71 ± 1.56	86.21 ± 1.49	84.06 ± 1.59	82.74 ± 1.63	86.19 ± 1.51
Sampler fusion	83.81 ± 1.55	84.66 ± 1.18	80.98 ± 1.10	79.25 ± 1.31	84.31 ± 1.17
DRW sampler fusion	84.49 ± 1.67	86.10 ± 1.16	82.12 ± 1.70	80.74 ± 1.31	86.21 ± 1.34
Proposed method	90.23 ± 1.27	89.61 ± 1.13	89.10 ± 1.35	88.46 ± 1.06	89.59 ± 1.68

**Table 3:** Sensitivity analysis of different target sequence lengths. Values are reported as mean ± standard deviation of five seed-level cross-validation averages. All metrics are presented on a 0–100 scale.

Target Sequence Length	Accuracy	Balanced Accuracy	Macro F1	Macro AUC	Average Precision	Macro Recall
2048	88.72 ± 1.35	87.82 ± 1.34	87.27 ± 1.71	92.87 ± 1.66	86.75 ± 1.59	87.81 ± 1.27
4096	90.23 ± 1.27	89.61 ± 1.13	89.10 ± 1.35	94.43 ± 1.42	88.46 ± 1.06	89.59 ± 1.68
8192	89.17 ± 1.50	88.44 ± 1.77	87.95 ± 1.49	93.49 ± 1.34	87.36 ± 1.48	88.42 ± 1.78

**Table 4:** Independent cross-dataset evaluation on the PhysioNet Gait in Parkinson’s Disease dataset. Values are reported as mean ± standard deviation across five repeated seeds. All metrics are presented on a 0–100 scale.

Dataset	Subjects Used	Severity Groups	Recording Modality	Accuracy	Balanced Accuracy	Macro F1	Macro AUC	Macro Recall
PhysioNet Gait in Parkinson’s Disease	93 PD subjects	M-M, *n* = 83; Moderate, *n* = 10; Mild, *n* = 0	Vertical ground reaction force, 100 Hz	84.34 ± 1.82	82.13 ± 1.92	81.68 ± 1.98	88.93 ± 2.14	82.12 ± 1.71

**Table 5:** Computational complexity and inference efficiency of the deep learning models.

Model	Input Used for Profiling	Trainable Parameters	FLOPs per Subject-Level Sample	Inference Time
1D-CNN	66 × 4096	0.47 M	1.36 GFLOPs	1.74 ± 0.12 ms
BiLSTM	66 × 4096	0.83 M	3.72 GFLOPs	3.89 ± 0.20 ms
Proposed Method	2 × 66 × 4096	1.12 M	2.86 GFLOPs	2.91 ± 0.17 ms

## Data Availability

The data analyzed in this study were derived from the publicly available WearGait-PD dataset, which is available in Synapse under DOI 10.7303/syn52540892 and described in Anderson et al. [11]. The external validation dataset used in this study was the public PhysioNet Gait in Parkinson’s Disease dataset [39,40], version 1.0.0, available at https://doi.org/10.13026/C24H3N under the Open Data Commons Attribution License v1.0. The processed results generated during the current study are available from the corresponding author upon reasonable request.
